# Comparative modeling and docking studies of p16ink4/Cyclin D1/Rb pathway genes in lung cancer revealed functionally interactive residue of RB1 and its functional partner E2F1

**DOI:** 10.1186/1742-4682-10-1

**Published:** 2013-01-01

**Authors:** Syeda Naqsh e Zahra, Naureen Aslam Khattak, Asif Mir

**Affiliations:** 1 Department of Bioinformatics and Biotechnology, International Islamic University, Islamabad, Pakistan; 2 Institute of Biochemistry and Biotechnology, Department of Biochemistry, Arid Agriculture University Rawalpindi, Rawalpindi, Pakistan

## Abstract

**Background:**

Lung cancer is the major cause of mortality worldwide. Major signalling pathways that could play significant role in lung cancer therapy include (1) Growth promoting pathways (Epidermal Growth Factor Receptor/Ras/ PhosphatidylInositol 3-Kinase) (2) Growth inhibitory pathways (*p53/Rb/P14ARF, STK11*) (3) Apoptotic pathways (*Bcl-2/Bax/Fas/FasL*). *Insilico* strategy was implemented to solve the mystery behind selected lung cancer pathway by applying comparative modeling and molecular docking studies.

**Results:**

YASARA [v 12.4.1] was utilized to predict structural models of *P16-INK4* and *RB1* genes using template 4ELJ-A and 1MX6-B respectively. WHAT CHECK evaluation tool demonstrated overall quality of predicted P16-INK4 and RB1 with Z-score of −0.132 and −0.007 respectively which showed a strong indication of reliable structure prediction. Protein-protein interactions were explored by utilizing STRING server, illustrated that *CDK4* and *E2F1* showed strong interaction with *P16-INK4* and *RB1* based on confidence score of 0.999 and 0.999 respectively. In order to facilitate a comprehensive understanding of the complex interactions between candidate genes with their functional interactors, GRAMM-X server was used. Protein-protein docking investigation of *P16-INK4* revealed four ionic bonds illustrating Arg47, Arg80,Cys72 and Met1 residues as actively participating in interactions with *CDK4* while docking results of *RB1* showed four hydrogen bonds involving Glu864, Ser567, Asp36 and Arg861 residues which interact strongly with its respective functional interactor *E2F1*.

**Conclusion:**

This research may provide a basis for understanding biological insights of *P16-INK4* and *RB1* proteins which will be helpful in future to design a suitable drug to inhibit the disease pathogenesis as we have determined the interacting amino acids which can be targeted in order to design a ligand *in-vitro* to propose a drug for clinical trials. Protein -protein docking of candidate genes and their important interacting residues likely to be provide a gateway for developing computer aided drug designing.

## Background

Lung cancer is the most prevalent type of cancer which causes greater than millions worldwide cancer-related death [1,2]. About 85−90% of lung cancer is caused due to tobacco smoking resulting in bronchogenic carcinoma [3,4].

It has been classified into four distinct histological types, namely, small cell lung carcinoma (SCLC) and three non-small cell lung carcinoma (NSCLC) types; adenocarcinoma (ADC), squamous cell carcinoma (SQC), and large cell carcinoma (LCC) [5]. This type of cancer develops its proliferation through alterations in oncogenes, such as EGFR and tumor suppressor genes, such as *TP53, RB1, CDKN2A/p16*[1,6]. Smoking is the most important root of all lung cancer types but small-cell lung cancer and squamous-cell carcinoma are more strongly caused by tobacco smoke. However, in patients who have never smoked in their life, adenocarcinoma is the most frequent type.

Epigenetic changes have also a profound impact in development of lung cancer. In the DNA promoter sequence of protein-coding genes, hypermethylation of cytosine in clusters of CpG dinucleotides can cause loss of gene expression. Research indicated that more than 80 genes are hypermethylated including tumour suppressor genes, e.g. p16INK4a in this type of cancer. Early detection of methylated DNA in sputum or blood of a patient can be an effective biomarker for diagnosis of lung cancer at initial stages. DNA promotor methylation and histone deacetylation are reversible processes; therefore, pharmacological inhibition can be used as therapeutic strategy to cure this disorder as this strategy may reverse gene silencing which will be beneficial in curing lung cancer [7].

Several different signalling pathways play significant roles in lung cancer therapy, for example, Growth promoting pathways (Epidermal Growth Factor Receptor/Ras/ PhosphatidylInositol 3-Kinase),Growth inhibitory pathways (*p53/Rb/P14ARF, STK11*), Apoptotic pathways (*Bcl-2/Bax/Fas/FasL*),DNA repair and immortalisation genes. Among these pathways, we have selected *p16INK4/cyclin D1/Rb* pathway for this particular study.

Expression profiling of eleven genes involved in this pathway was done by utilizing several databases like BioGPS, HPRD and GeneCards. Two candidate genes were short listed based on (i) Molecular Function, (ii) Biological process and (iii) Cellular location. Furthermore, common functional partners of selected pathway genes through STRING database were evaluated and it was found that three dimensional structures of these short listed proteins *P16-INK4A* and *RB1* are not reported to have been resolved yet. Therefore, in current study, 3-D structures are predicted using a computational methodology i.e., homology modeling. Furthermore, Protein-protein docking was performed for proteins encoded by these genes.

## Results

Templates selected for all proteins with optimal alignment of fist template and good alignment for remaining templates sorted by their overall quality Z-scores and E-values are listed in Table 1.

**Table 1:** Templates sorted by their overall quality Z-scores and E-values

**Protein**	**Model ID**	**Z-Score**	**Alignment**	**BLAST E-value**
*RB1*	4ELJ-A	−0.007	Good	0
*P16-INK4A*	1MX6-B	−0.289	Optimal	7e-032
	1BLX-B	−0.528	Good	1e-034
	1BD8-A	−0.620	Good	2e-036
	1BI7-B	−1.084	Good	6e-042
	2A5E-A	−2.110	Good	9e-050

**Figure 1:**
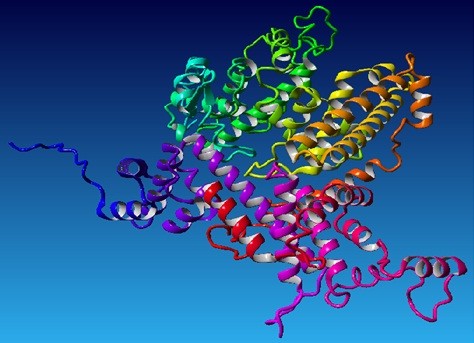
**Predicted Structure of RB1 using 4ELJ template with Z-Score of −0.007.** Red color represent alpha helix, cyan color represents beta sheets and white represents loops.

**Figure 2:**
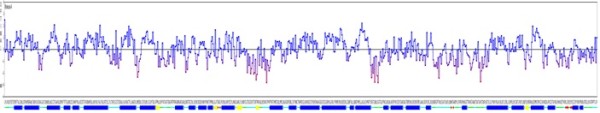
Overall quality of predicted RB1 model.

**Figure 3:**
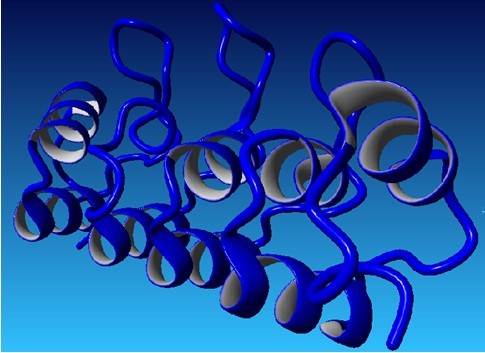
**Predicted Structure of P16-INK4A using 1MX6 template with Z-Score of −0.289.** Red color represents alpha helix and loops are shown in white.

**Figure 4:**
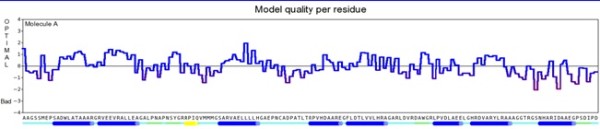
Overall quality of predicted P16-INK4A model.

Hybrid structure of *RB1* protein was generated using best aligned parts of templates (Figure 1). Among the selected templates for RB1, 4ELJ was best scoring template used for modeling. Plot of its overall quality Z-score, shown per residue is displayed in Figure 2. For *P16-INK4A* protein, hybrid structure was generated using best aligned parts of all the five templates (Figure 3). The best scoring template used for modeling was 1MX6. Plot of its overall quality Z-score, shown per residue is displayed in Figure 4.

### Protein-protein docking

**Figure 5:**
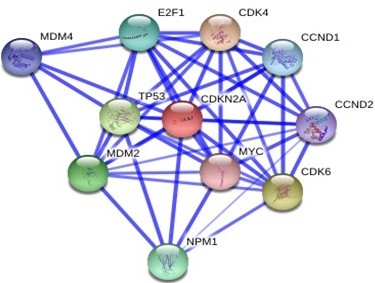
Functional partners of P16-INK4A protein through STRING database.

**Figure 6:**
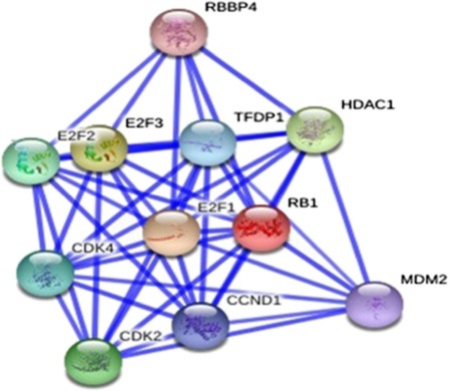
Functional partners of RB1 protein through STRING database.

**Figure 7:**
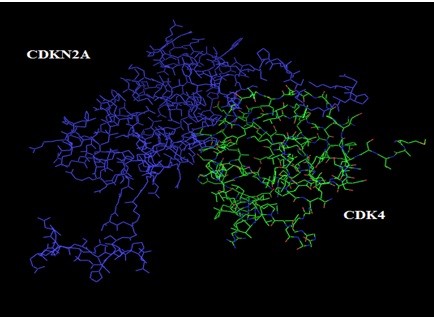
Docked complex of P16-INK4A and CDK4 showing P16-INK4A in blue and CDK4 in green.

**Figure 8:**
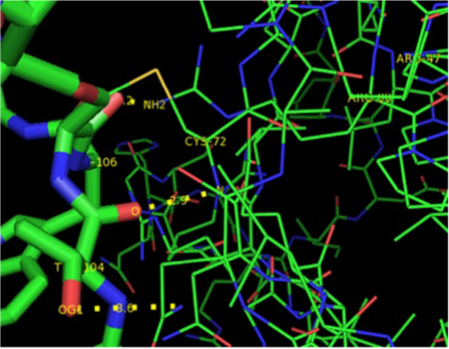
**Interactions between P16-INK4A and CDK4.** P16-INK4A is shown in lines and CDK4 in sticks format.

**Figure 9:**
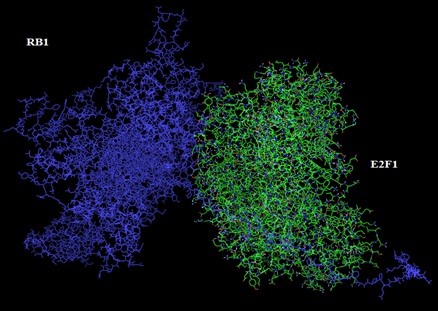
Docked Complex of RB1 and E2F1showing RB1 in blue and E2F1 in green.

**Figure 10:**
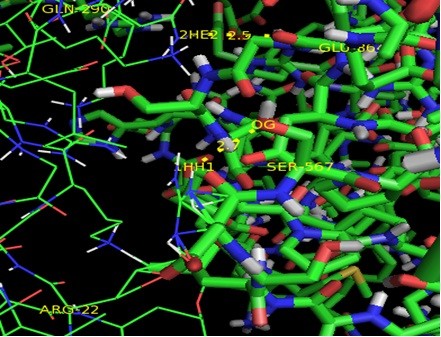
Interactions between RB1 and E2F1 showing RB1 in lines and E2F1 in sticks format.

GRAMM-X was utilized for protein-protein docking of two proteins *RB1* and *P16-INK4A* for which no ligand was reported in literature/databases. Figure 5 and 6 shows the functional partners for these proteins obtained through STRING database. Table 2 shows the functional proteins which are found to be common between *RB1* and *P16-INK4A.* Table 3 displays the protein and their functional interactors considered for docking. Table 4 shows the GRAMM-X docking results and Figure 7, 8, 9 and 10 displays the docked complexes and their interactions.

**Table 2:** Common functional partners between RB1 and P16-INK4A

**Proteins**	**Common functional partners**
*RB1* and *P16-INK4A*	CDK4
	E2F1
	MDM2
	CCND1

**Table 3:** Proteins and interactors for protein-protein docking

**Receptor protein**	**Functional interactors**
***P16-INK4A***	CDK4
***Rb1***	E2F1

**Table 4:** **Binding interactions for *Rb1 *and *P16-INK4A***

**Receptor protein**	**Interacting protein**	**Interactions (Receptor residue →Interacting protein residue)**	**Bond distance**
***P16-INK4A***	*CDK4*	Arg47:NH2 →Thr 104:OG1	3.6
		Arg 80:NH2 → Trp 106: O	2.2
		Met 1: N →Val 9: O	3.4
		Cys72:N →Thr104:O	2.9
***RB1***	*E2F1*	Glu 864:O →Gln 290:2HEZ	2.5
		Ser 567:OG →Arg 22:1HH1	2.7
		Asp 36: OD2 →Lys 89:1HZ	2.2
		Arg 861:2HHZ →Thr 285:OG1	1.9

## Discussion

In current study, 3D structures of the prioritized genes are predicted. *RB1*and *P16-INK4A* are found to have expressions in lung tissue. Best docking complex of *RB1* and *E2F1* analysis suggested that , hydrogen bond interactions are found between O of Glu 864, Ser 567, Asp 36, Arg 861(of *RB1*) and H of Gln 290, Arg 22,Lys 89 and Thr 285 (of *E2F1*) with bond distances of 2.5,2.7,2.2 and 1.9 respectively. *P16-INK4A* and *CDK4* protein-protein complex showed ionic bond interactions between Arg47, Arg80, Met 1, Cys72, Thr 104, Trp 106 and Val9 with 3.6, 2.2, 2.9 and 3.4 bond distances indicating the potential role of these residues in protein-protein interaction. Interactions of *RB1* and *E2F1* complexes will help in cell cycle arrest in G1 phase as *RB1* acts as a transcription repressor of E2F1 target genes. The underphosphorylated, active form of RB1 interacts with E2F1 and represses its transcription activity, leading to cell cycle arrest. *P16-INK4A* and *CDK4* interactions help to inhibit the proliferation of the cells . Results revealed through Protein-protein binding may provide a basis for designing a suitable drug for preventing this widely spreading disease by using the information retrieved about the amino acids involved in interactions with the respective proteins.

## Conclusion

3-dimensional structure prediction of most plausible candidate genes proposed that it may be used further to understand the potential mechanism of lung cancer development and role of these proteins in causing abnormalities. By exploring protein- protein docking interaction with in wild type and mutant protein can open the new gate for computer aided drug designing for the better identification of potential drug inhibitor.

## Materials & methods

### Sequence retrieval and 3d model building

Sequences in FASTA format of *P16-INK4* and *RB1* were retrieved from NCBI (National Centre of Biotechnology Information) having accession numbers of P42771, P06400 and OMIM id’s of 614041 and 600160 respectively. Since the target sequence was the only available information, possible templates were identified by running 3 PSI-BLAST iterations to search the PDB for match (i.e. hits with an E-value below the homology modeling cutoff 0.5).

Comparative modeling approach was implemented to generate 3D structures of genes using YASARA software. YASARA generated a hybrid structure using 2–5 templates which are ranked on the basis of alignment score (PSI_BLAST) and structural quality (Z_Score) according to WHAT CHECK [8] obtained from the PDBFinder2 [8] database for all six candidate genes. Selected Parameters used by YASARA for structure prediction are mentioned in Table 5.

**Table 5:** Parameters selected for YASARA comparative modeling

**Parameters**	**Value**
**PSI-Blast iteration**	3
**Psi-BLAST E-value**	0.5
**Oligomerization state**	4
**Templates**	2–5
**Alignment per template**	5
**Modeling Speed**	Slow
**Loop Samples**	50

### Model validation

YASARA softwares uses WHAT CHECK [8] obtained from the PDBFinder2 [8] database for generating plot of overall quality Z-score.

### Molecular docking

Protein-protein docking of *P16-INK4* and *RB1* was carried out through GRAMM-X docking web server.

### Protein-protein docking

Protein to be used as a ligand in protein-protein docking was retrieved from STRING database, an online database for physical (direct) and functional (indirect) protein–protein interactions [9] and its 3D structure was predicted using ab-initio approach through I-TASSER server. GRAMM-X docking server [10] was used for Protein-protein docking which generated a docked complex. Post docking analysis was carried out using Pymol software which is a molecular visualization system for use in structural biology which provides a user with high quality 3D images of small molecules and biological macromolecules, such as proteins.

## Competing interests

The authors declare that they have no competing interests.

## Authors’ contributions

The work presented here was carried out in collaboration between all authors. AM and NAK defined the research theme and designed methods analyzed the data, interpreted the results and wrote the paper. NZ carried out all the work and analysis of results under the guidance of NAK. AM also provided suggestions to the interpretation of results. All authors have contributed to, seen, read and approved the manuscript.
